# Scale development to evaluate differences between concern about falling and fear of falling: the concern and fear of falling evaluation

**DOI:** 10.3389/fpsyg.2024.1336078

**Published:** 2024-01-22

**Authors:** Taylor N. Takla, Patricia N. Matsuda, Tracy E. Herring, Ana M. Daugherty, Nora E. Fritz

**Affiliations:** ^1^Neuroimaging and Neurorehabilitation Laboratory, Wayne State University, Detroit, MI, United States; ^2^Translational Neuroscience Program, Wayne State University, Detroit, MI, United States; ^3^Department of Rehabilitation Medicine, Division of Physical Therapy, University of Washington, Seattle, WA, United States; ^4^Department of Rehabilitation Medicine, University of Washington, Seattle, WA, United States; ^5^Department of Psychology, Wayne State University, Detroit, MI, United States; ^6^Institute of Gerontology, Wayne State University, Detroit, MI, United States; ^7^Department of Health Care Sciences, Wayne State University, Detroit, MI, United States; ^8^Department of Neurology, Wayne State University, Detroit, MI, United States

**Keywords:** fall, fear of falling, multiple sclerosis, concern about falling, scale development

## Abstract

**Purpose:**

Individuals with multiple sclerosis (MS) experience fear of falling (FOF), which is associated with negative health and quality-of-life consequences. Prior research has used FOF and concern about falling (CAF) interchangeably, but persons with MS report that CAF and FOF represent separate constructs that lie on a continuum. Unfortunately, no scale exists to understand the differences between CAF and FOF. Therefore, we developed a novel questionnaire, the Concern and Fear of Falling Evaluation (CAFFE), in which respondents rank their CAF and FOF on a continuum across various activities. This study aims to describe the scale development process and examine its psychometric properties.

**Methods:**

In a single online survey, MS participants responded to demographic questionnaires, indicated whether they experience CAF and FOF, and completed the CAFFE. Psychometric evaluation of the CAFFE involved internal consistency, split-half cross validation, exploratory factor analysis (EFA), and confirmatory factor analysis (CFA).

**Results:**

Out of 1,025 respondents, 64.6% reported CAF and 47.2% reported FOF. The EFA yielded a two-factor solution encompassing activities in open (factor 1) and closed environments (factor 2). The CFA replicated this two-factor solution and the CAFFE demonstrated excellent internal consistency (α = 0.98).

**Conclusion:**

The 27-item CAFFE is a highly reliable and valid measure capturing the tipping point at which point CAF moves to FOF. Future research should seek to define the tipping point from the MS community, as CAF may be an adaptive mechanism, whereas FOF may be a maladaptive behavior.

## 1 Introduction

**Figure 1:**
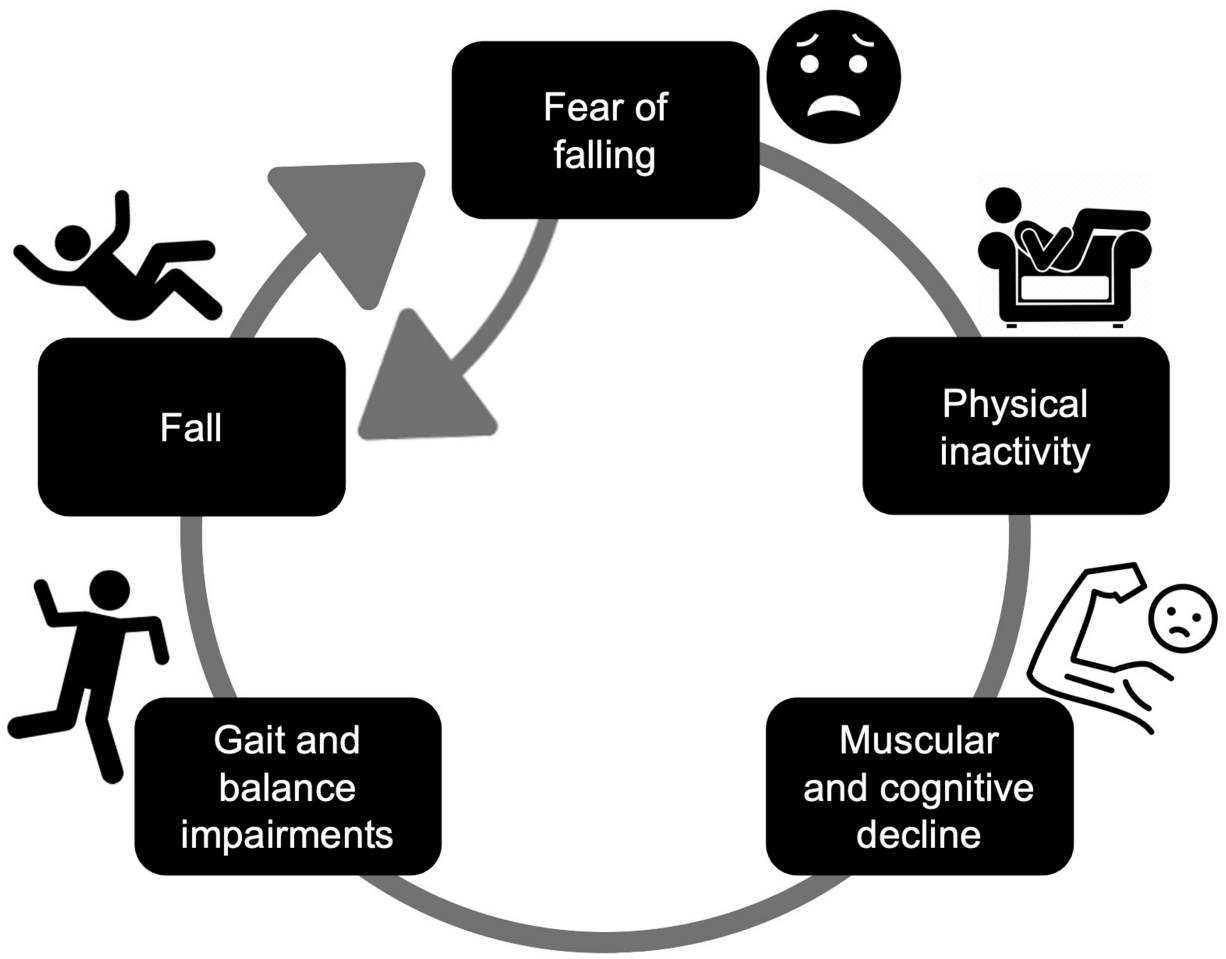
Consequences of FOF leading to a vicious cycle of inactivity, functional decline, and falls.

Multiple sclerosis (MS) is a chronic neurodegenerative disease marked by inflammatory lesions in the central nervous system resulting in demyelination, axonal degradation, and neurological dysfunction (Bjartmar and Trapp, 2001). As a result, individuals with MS experience a variety of psychological, cognitive, and motor symptoms (Beiske et al., 2008; Fritz et al., 2022); some of the most common and debilitating impairments include balance deficits and walking difficulty (Kelleher et al., 2010). Consequently, falls are extremely common among persons with MS, with over 50% of this population experiencing a fall within any six-month period (Matsuda et al., 2011; Gunn et al., 2014). An important contributor and consequence of falling is fear of falling (FOF) which is reported by more than 60% of the MS community (Scholz et al., 2021). FOF results in a downward spiral of inactivity and social isolation, muscular and cognitive decline, and subsequently, increased fall risk (Peterson et al., 2007; Mazumder et al., 2014). Individuals with a fall history report FOF significantly more than those who have not fallen (Kalron and Achiron, 2013; Lavedán et al., 2018). Surprisingly, nearly 20% of individuals with MS who have not fallen in the past year still report FOF (Kalron and Allali, 2017). Prior research has identified FOF as an independent predictor of future falls (Friedman et al., 2002; Mazumder et al., 2015). Therefore, individuals become trapped in a vicious cycle (Figure 1), beginning with either the development of FOF or the experience of a fall, leading to physical inactivity, muscular weakness, cognitive decline, gait and balance impairments, and subsequent increased frequency of falls and greater FOF. Though interventions are critically needed to break this cycle, FOF must be better defined before effective strategies targeting this fear can be developed for individuals with MS.

Current FOF research often uses concern as a proxy for fear, defining FOF as a lasting concern about falling that leads to an individual avoiding activities that they are capable of performing (Tinetti and Powell, 1993). However, asking individuals about their concern about falling (CAF) may not truly capture their fear, because persons with MS experience concern separate from fear, but also report that these constructs lie on a continuum so that at a certain point, higher concern accompanies the beginning feelings of fear (Matsuda and Hoffman, 2022). Unfortunately, no scale exists to understand the continuum between CAF and FOF. The Falls Efficacy Scale International (FES-I) is the most frequently used measure of FOF in both MS (Scholz et al., 2021) and elderly populations (Whipple et al., 2018). The FES-I has individuals rank their *concern* about falling (i.e., 1 = not concerned at all, 4 = very concerned) for various activities of daily living (Yardley et al., 2005). Thus, the FES-I may measure concern rather than fear. An assessment that places CAF and FOF on a continuum to reflect views of persons with MS is critically needed to understand the differences and effects of these two constructs. It is possible that CAF represents an adaptive, appropriate avoidance of activities when an individual is at a high risk for falling, whereas FOF may represent a maladaptive, inappropriate avoidance of activities that the individual is fully capable of performing. Moreover, understanding how CAF and FOF may vary in different contexts is clinically relevant for identifying specific environmental factors that contribute to the manifestation and exacerbation of these psychological constructs. This would enable clinicians to develop targeted strategies for FOF and fall prevention across specific contexts.

Therefore, the objective of this study is to examine CAF and FOF through different contexts in persons with MS. To achieve this objective, we developed a novel questionnaire, the Concern and Fear of Falling Evaluation (CAFFE), in which respondents rank their CAF and FOF on a continuum in different environmental contexts. The CAFFE is not intended to replace the FES-I, which only assesses CAF, or other assessments [e.g., Activities-Specific Balance Confidence Scale (Powell and Myers, 1995), Fear of Falling Avoidance Behavior Questionnaire (Landers et al., 2011), etc.], but rather to compliment other assessments and to provide a more comprehensive understanding of CAF and FOF. The purpose of this study is to describe the scale development process (Boateng et al., 2018), examine its psychometric properties, and to identify a tipping point in which CAF moves to FOF. We hypothesized that CAF would be more prevalent than FOF, and that the CAFFE would be a reliable and valid measure of CAF and FOF in different contexts, with a defined tipping point at which concern turns into fear.

## 2 Materials and methods

A sample of 1,025 individuals with MS responded to the online survey distributed via email though the National MS Society, which was available from September 6 through October 3, 2022. Inclusion criteria required participants to be at least 18 years old, have a self-reported diagnosis of any type of MS, be able to read and write in English, and consent to the survey. The survey consisted of demographic information, MS subtype, questions asking whether respondents experience CAF or FOF (answer choices being yes, maybe, or no), and the CAFFE.

### 2.1 Concern and fear of falling evaluation

**Figure 2:**
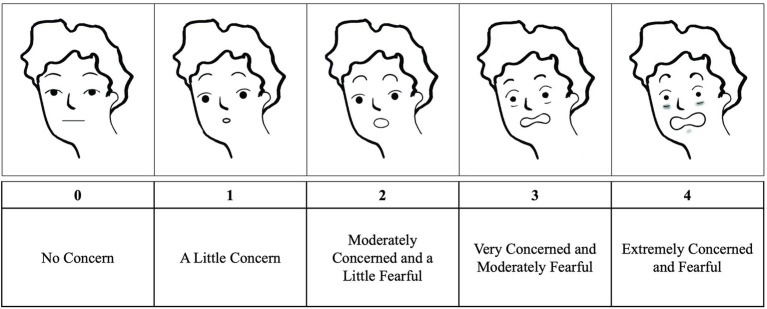
Concern about falling and fear of falling continuum scale with corresponding faces. (Adapted from Raccanello et al., 2017).

The CAFFE is a self-report measure developed by our research team to evaluate contexts for CAF and FOF. Our research team created content items consisting of common scenarios and contexts where patients experience falls or report experiencing FOF that reflect our shared clinical experience. Based on those criteria, we developed 28 items representing transferring positions, dynamic balance tasks, activities of daily living, walking through different environments, walking on different surfaces, and attending different events (Table 1). The questionnaire asks respondents to rate their CAF and FOF in different contexts for 28 activities. As persons with MS experience CAF and FOF on a continuum, where at high levels of concern, there is an addition of fear, we developed a 5-point scale consisting of anchor items that correspond to patients’ experiences. Respondents use a 5-point Likert-type scale that corresponded to faces depicting concern and fear (Figure 2), where 0 = no concern, 1 = a little concern, 2 = moderately concerned and a little fearful, 3 = very concerned and moderately fearful, and 4 = extremely concerned and fearful. To reflect patients’ perspectives, the scale is structured as a continuum in which fear exists in addition to concern, so that at the midpoint of the scale at moderate concern anchor, there is an experience of fear as well. These constructs are entirely dependent, in the sense that you cannot be fearful without being concerned. Responses to the individual items represents an individual’s level of concern or the addition of fear in specific contexts. Participants were given the following instructions: “Please use this scale to rate the following activities. Even if you do not regularly perform this activity, try and imagine how you would feel if you had to do the activity. If you normally use a walking aid to do the activity or hold onto someone, rate your confidence as if you were using these supports.”

**Table 1:** Initial list of the concern and fear of falling evaluation (CAFFE) items.

Item No.	Activity
1	Moving from sit to stand or stand to sit
2	Moving from a chair to bed or bed to chair
3	Picking up something off the floor
4	Reaching for something overhead
5	Cleaning your home (e.g., dusting, sweeping, etc.)
6	Getting dressed and/or undressed
7	Taking a bath or shower
8	Cooking or preparing a meal
9	Walking in my home
10	Walking up and down stairs
11	Walking in my neighborhood
12	Walking on uneven ground/sidewalks
13	Walking up/down a ramp
14	Walking or going out at night
15	Walking long distances (10 city blocks, approximately 1 mile)
16	Crossing the street with a stoplight
17	Crossing the street with a stop sign
18	Going out to the local market
19	Going out to the shopping mall
20	Going up/down the escalator
21	Walking in a crowd
22	Walking in a busy and noisy environment
23	Walking in a place that is unfamiliar or new
24	Going to a social event or party
25	Attending a sporting event
26	Walking in sunny/hot weather
27	Walking in rainy weather
28	Walking in icy/snowy weather

### 2.2 Statistical analysis

All statistical analyses were performed using IBM SPSS Statistics V 29.0.0.0. The sample of 1,025 individuals was randomly split into two halves, stratified by Patient Determined Disease Steps (PDDS) (Hohol et al., 1999) and MS subtype to cross-validate the CAFFE. Half of the sample (n_1_) was used to perform an exploratory factor analysis (EFA) to test a possible factor structure based on the nature of the items, and to review evidence to retain or reject items based on factor structure. Following common practice in EFA, items that equally cross-loaded onto 2 or more factors, or those with negligible loadings (<|0.3|) were reviewed for potential rejection. The EFA was fit with promax rotation to account for the ordinal Likert-scale item responses. Following EFA, internal scale consistency of each factor was assessed for internal consistency, and items were reviewed for potential exclusion based on poor reliability. The factor structure and scale reliability were tested for replication in the hold-out sample (n_2_) for validation of the CAFFE.

To examine the tipping point between CAF and FOF on the CAFFE, two subgroups were formed from the full sample: individuals who responded ‘yes’ to experiencing both FOF and CAF (*n* = 433, the concerned + fearful group) and individuals who only responded ‘yes’ to experiencing CAF and ‘no’ to FOF (*n* = 135, the concerned only group) on independent items not included in the CAFFE scale. Median summary scores were computed for each extracted factor, representing the median value for an individual’s responses to all items that identified the respective factor. In other words, summary scores were calculated by taking an individual’s cumulative responses to all items that fell into a respective factor and calculating the median score. The summary scores from each factor were used as independent variables in a binomial logistic regression to predict group membership into either the concerned + fearful group or the concerned only group, controlling for age and sex. Parameters of the model are reported for model significance with chi-square testing and Nagelkerke’s formula to approximate R^2^, and each unique predictor effect is reported with unstandardized b-weights and Wald chi-square significance testing (*W*) and corresponding odds ratio (OR) with the concerned only group set as the reference. To find the tipping point in the scale, the b-weights from each summary score were used in a simple slopes analysis to predict the probability of being an individual that reported FOF using a cutoff of 70% or greater likelihood based on estimated odds ratios.

## 3 Results

In total, 1,025 individuals with MS (815 females, 210 males, aged 20–89 years) completed the survey. For a complete demographic profile of the sample, see Table 2. On average, the total completion time for all 27 items in the CAFFE was approximately 4 min.

**Table 2:** Sample description.

Variable	Descriptive statistic
Sample size	1,025
Female, *n* (%)	815 (79.5%)
Age (years)	54.08 ± 12.55
MS subtype, *n* (%)	Relapsing–remitting MS: 695 (67.8%) Progressive MS: 301 (29.4%) Unknown: 29 (2.8%)
Disease severity (PDDS)	3.01 ± 2.20
Race, *n* (%)	White: 883 (86.1%) Black or African American: 77 (7.5%) Hispanic or Latino: 33 (3.2%) Asian or Pacific Islander: 8 (0.8%) Native American or Alaskan Native: 4 (0.4%) Biracial or multiracial: 12 (1.2%) Other: 8 (0.8%)
Disease Modifying Therapy, *n* (%)	777 (75.8%)
Have Fallen in the Past Month, *n* (%)	381 (37.2%)
Assistive Device Use Inside, *n* (%)	301 (29.0%)
Assistive Device Use Outside, *n* (%)	481 (46.3%)

Demographic profile of the sample who responded to the online survey. Sample means and standard deviations are reported (M ± SD); Patient Determined Disease Steps (PDDS).

### 3.1 CAF and FOF prevalence

Overall, 617 individuals (60.2%) reported that CAF is not the same as FOF. A greater percentage of the sample responded “yes” to experiencing CAF compared to FOF. Interestingly, a similar number of people responded “maybe” and “I do not know” to experiencing CAF and FOF (Table 3).

**Table 3:** Prevalence of concern about falling and fear of falling.

Responses	Concern about falling	Fear of falling
No, *n* (%)	190 (18.5%)	355 (34.6%)
I do not know, *n* (%)	9 (0.9%)	14 (1.4%)
Maybe, *n* (%)	164 (16.0%)	172 (16.8%)
Yes, *n* (%)	662 (64.6%)	484 (47.2%)

### 3.2 Scale development: exploratory factor analysis and internal consistency of the CAFFE

To explore the underlying factor structure of the CAFFE, all 28 items in the questionnaire were submitted to an EFA with Promax rotation. The EFA resulted in the extraction of two factors. Review of the factor loadings identified one item for exclusion: the stairs item (item #10) did not load well into either factor (factor 1 loading = 0.544; factor 2 loading = 0.329). Thus, this item was removed, and the factor analysis was repeated with the remaining leaving 27 items. The Keiser-Meyer-Olkin value (KMO = 0.973) and Bartlett’s test of sphericity (χ^2^ (351) = 16,078.22, *p* < 0.001) indicated the data and sample size were sufficient for factor analysis. The EFA resulted in a two-factor solution as the best fit for the data. Factor 1 was comprised of 18 items (#11–28 from Table 1) with factor loadings from 0.904 to 0.643 (Table 4). Factor 2 was comprised of 9 items (#1–9 from Table 1) with factor loadings from .899 to .550 (Table 4). Viewing the structure that the data confirmed, it appeared the two factors represent two different contexts. Review of the content of items in factor 1 reflected attributes of open environments (contexts outside of the home), including activities in public spaces, outdoors, unfamiliar environments, and activities impacted by weather. Review of the content of items in factor 2 reflected attributes of closed environments (contexts within the home), including indoor, enclosed activities and familiar environments. Thus, the two extracted factors represent contexts of self-reported CAF and FOF. Together, these factors explained 70.32% of the variance in responses. The overall questionnaire (27 items) demonstrated excellent internal consistency, α = 0.98, as well as each factor: factor 1 α = 0.98 and factor 2 α = 0.94.

**Table 4:** Exploratory and confirmatory factor analysis of the items of the CAFFE.

	Exploratory factor analysis of the items of the CAFFE		Confirmatory factor analysis of items of the CAFFE		
Items	Factor		Factor		Environment
	**1**	**2**	**1**	**2**	Activities outside of the home (open environments)
Walking in a place that is unfamiliar or new	**0.904**	0.014	**0.932**	−0.048	
Walking in a busy and noisy environment	**0.892**	−0.004	**0.941**	−0.068	
Walking long distances (10 city blocks, approximately 1 mile)	**0.882**	−0.012	**0.727**	0.153	
Attending a sporting event	**0.880**	−0.011	**0.940**	−0.095	
Walking in a crowd	**0.877**	0.037	**0.956**	−0.066	
Walking in icy/snowy weather	**0.873**	−0.053	**0.734**	0.078	
Walking in rainy weather	**0.860**	−0.016	**0.735**	0.103	
Crossing the street with a stoplight	**0.811**	0.091	**0.793**	0.087	
Going to a social event or party	**0.808**	0.060	**0.843**	−0.008	
Crossing the street with a stop sign	**0.803**	0.087	**0.798**	0.092	
Walking on uneven ground/sidewalks	**0.797**	0.067	**0.653**	0.246	
Walking or going out at night	**0.796**	0.050	**0.731**	0.151	
Going out to the shopping mall	**0.796**	0.125	**0.768**	0.127	
Walking in my neighborhood	**0.753**	0.159	**0.680**	0.219	
Going out to the local market	**0.698**	0.197	**0.687**	0.210	
Going up/down the escalator	**0.697**	0.105	**0.701**	0.078	
Walking in sunny/hot weather	**0.683**	0.098	**0.584**	0.150	
Walking up/down a ramp	**0.643**	0.237	**0.561**	0.317	
Moving from a chair to bed or bed to chair	−0.090	**0.899**	−0.104	**0.909**	Activities inside of the home (closed environments)
Moving from sit to stand or stand to sit	−0.040	**0.842**	0.005	**0.780**	
Getting dressed and/or undressed	−0.090	**0.806**	−0.053	**0.738**	
Picking up something off the floor	0.126	**0.723**	0.045	**0.756**	
Cooking or preparing a meal	0.141	**0.708**	0.296	**0.544**	
Reaching for something overhead	0.211	**0.611**	0.201	**0.550**	
Taking a bath or shower	0.191	**0.604**	0.112	**0.678**	
Cleaning your home (e.g., dusting, sweeping, etc.)	0.278	**0.586**	0.304	**0.534**	
Walking in my home	0.281	**0.550**	0.175	**0.632**	

The items go in order of their factor loadings from greatest to least for the EFA, but not for the CFA. However, the items that fell into their respective category remained the same for both factor analyses. The bolded values represent which factor the item loaded into.

### 3.3 Scale validation: confirmatory factor analysis and internal consistency with a hold-out sample

The confirmatory factor analysis using the hold-out sample resulted in similar findings. The sampling and correlation structure were adequate for the factor analysis (KMO = 0.971; Bartlett’s test of sphericity χ^2^ (351) = 15332.39, *p* < 0.001). The two-factor solution was replicated, accounting for 67.73% of the variance in responses in the hold-out sample. The same items identified each factor with similar loadings: factor 1 was comprised of the 18 items in open environments with loadings from 0.956 to 0.561, and factor 2 was comprised of the 9 items in closed environments with loadings from 0.909 to 0.534 (Table 4). The internal consistency of the CAFFE remained high, α = 0.98. The reliability of each factor remained high as well, factor 1 α = 0.98, factor 2 α = 0.93.

### 3.4 CAFFE responses predict independent reports of FOF: binomial logistic regression

A binomial logistic regression was to predict experiencing FOF by the summary scores from factor 1 (open environments) and factor 2 (closed environments), and age and sex as covariates. The summary scores represent an estimate of one’s CAF and FOF in different contexts by taking the median value across all items that fell either within the open environment factor (factor 1) or the closed environment factor (factor 2). The outcome was group membership of having reported only CAF (concerned only) or experiencing both concern and FOF (concerned + fearful). All assumptions of the modeling were met except for no outliers in continuous predictors; there were 9 outliers in the closed factor summary score.

The overall model was significant, χ^2^ (4, *N* = 568) = 93.76, *p* < 0.001, and accounted for 22.8% of the variability in the likelihood of experiencing FOF (Nagelkerke pseudo-*R*^2^ = 0.228). Age did not significantly predict differences in group membership (Table 5). Females were 2.46 times more likely to experience FOF as compared to males, which was significant (Table 5). Taking these covariates into account, higher responses on the open environment summary score corresponded to a 0.52 increase in the likelihood of reporting FOF (Table 5). This corresponds to every unit increase in the open environment score predicting a 40.54% increase in the likelihood of an individual being classified as concerned + fearful as compared to only concerned. Independent of this effect, higher closed environment summary scores also predicted increased likelihood of reporting FOF (Table 5). This corresponds to every unit increase on the closed environment scale predicting a 59.83% increase in the likelihood of an individual being classified as fearful + concerned as compared to only concerned. The overall accuracy of the model was 76.2%. The model was highly sensitive for classifying individuals who were concerned + fearful (95.8%), but poorly classified those who were only concerned (13.3%).

**Table 5:** Logistic regression results.

Source	*b*	Wald χ^2^	*p*	OR
Age	−0.003	0.108	0.742	0.98
Sex	0.90	12.74	< 0.001	2.46
Open environments	0.52	13.26	< 0.001	1.68
Closed environments	0.59	7.99	0.005	1.80

### 3.5 Identifying the tipping point from concern only to fear

**Figure 3:**
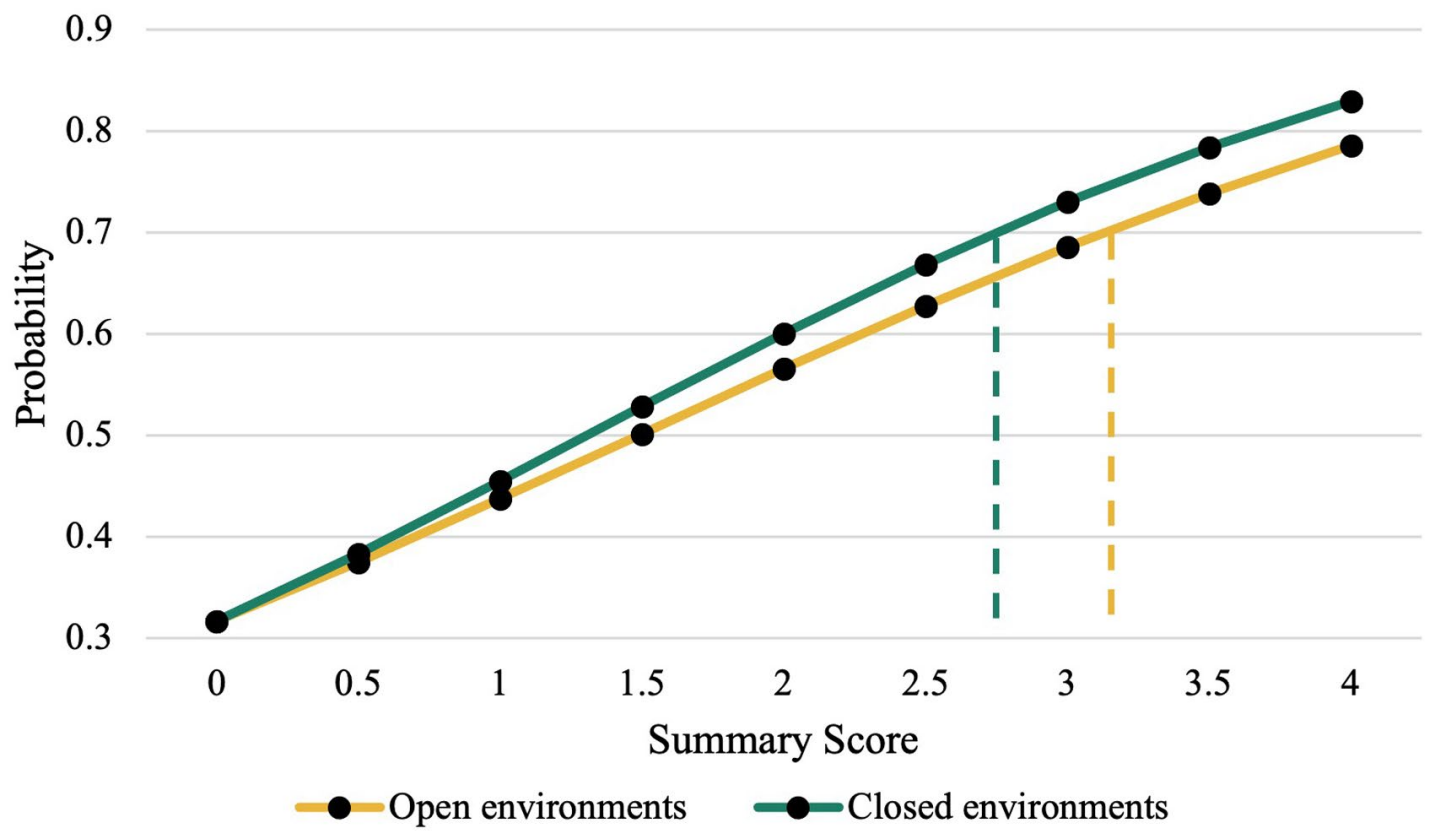
Simple slopes analysis. The tipping points for open (3.12) and closed (2.75) environments correspond to the summary scores at a probability of 0.7. A 0.7 probability cutoff was chosen to have 70% or better probability of correctly identifying individuals with FOF. The equation Y = e^u^/(1 + e^u^) was used, where u = −0.768 + .52x for the open environments, and u = −0.768 + .59x for the closed environments.

To find the tipping point between CAF and FOF in the CAFFE for both open and closed environment summary scores, the intercept from the logistic regression (−0.768) and the respective b-weights from each factor (open environments, *b* = 0.517; closed environments, *b* = 0.588), were used to predict the likelihood that an individual reported experiencing FOF on the independent question. At a minimal practical criterion of at least a 0.70 probability of correctly identifying people with FOF, the tipping point from concern to fear corresponded to a summary score of 3.12 for open environments, and 2.75 for closed environments (Figure 3).

## 4 Discussion

The current study evaluated CAF and FOF prevalence in the MS community and presented a new questionnaire, the CAFFE, that contains a defined tipping point at which CAF turns into FOF for both activities outside of the home (open environments) and within the home (closed environments). Our data suggests that CAF impacts a greater percentage of the MS community than FOF, that the CAFFE is a reliable and valid tool to measure CAF and FOF, and that by using the CAFFE, an individual could score lower across closed environment items (tipping point = 2.75) to be identified as experiencing FOF compared to open environment items (tipping point = 3.12).

We hypothesized that CAF would be more prevalent than FOF across the MS population. Our hypothesis was confirmed, as about two thirds of the sample responded “yes” to CAF, and just under half responded “yes” to FOF. Interestingly, about 16% of the sample responded “maybe” to experiencing CAF and FOF (Table 3), highlighting complexity of these concepts. It is possible that the cognitive impairments experienced by persons with MS may result in difficulty understanding these abstract constructs, potentially leading to confusion and misinterpretations. On the other hand, it may be that these participants lack awareness of their own thoughts regarding CAF and FOF, leading to uncertain self-reports. Consistent terminology is needed to define CAF and FOF in order for persons with MS to provide accurate responses to their feelings regarding these concepts. Generally, clinicians fail to ask patients with MS about both their CAF and FOF, which may contribute to the confusion when asked about them separately. Importantly, a previous study utilizing focus groups found that engaging in a group discussion about the differences between these two concepts enabled participants with MS to arrive at a more refined and comprehensive understanding of these psychological constructs (Matsuda and Hoffman, 2022).

A main objective of this study was to develop an easily administered questionnaire with sound psychometric properties that assesses and distinguishes between CAF and FOF. The average completion time (approximately 4 min) suggests that the CAFFE is a quick tool to evaluate CAF and FOF. An EFA revealed a two-factor solution, with activities outside of the home falling into one factor, and activities inside of the home falling into the other factor. Our results provide preliminary evidence that the CAFFE is a highly reliable and valid measure for evaluating CAF and FOF during daily activities in persons with MS. The large sample was split in half, stratified by disease-severity (PDDS) and MS subtype, to cross validate the CAFFE. The CAFFE demonstrated excellent reliability for both halves of the sample and for each factor separately. The validity of the CAFFE was supported by results from the CFA, replicating the two-factor solution, with the same items loading into each factor.

Additionally, we hypothesized that the CAFFE would have a defined tipping point at which concern turns into fear that would be in line with the personal experiences of persons with MS. Our results support a unique tipping point for each factor, with closed environments having a tipping point corresponding to a lower value on the original CAFFE scale (Figure 2) to meet the 0.7 probability cutoff than open environments. To our knowledge, no currently available scales have investigated this tipping point, or evaluated CAF and FOF as separate experiences that fall along a continuum. Therefore, the CAFFE provides clinicians and researchers with a quick instrument that is highly reliable and valid to assess CAF and FOF with defined tipping points in persons with MS.

Our data also showed that both open environment and closed environment summary scores were significant predictors of being classified as concerned + fearful. As either summary score increased, an individual was more likely to be classified as concerned + fearful because both odds ratios are greater than one. Interestingly, the open environment summary score had a lower odds ratio (1.68) compared to closed environments (1.80), suggesting that CAF and FOF in closed environments has a greater impact on identifying individuals who reported FOF compared to open environments. This finding is further emphasized when considering the unique tipping points for open and closed environments, as the closed environments summary score (2.75) met the 70% cutoff at a lower value than the open environment summary score (3.12). In other words, across the items in the CAFFE, concern and fear reported during activities inside of the home were more sensitive at classifying individuals as fearful of falling than activities outside of the home. Considering that the tipping points were context dependent, clinicians can use this information to evaluate FOF in specific environments. If an individual is scoring a 3 or 4 on their summary score in either context, it serves as a clear indicator of fear that may lead to maladaptive and avoidance behaviors. Clinicians can immediately use the information provided by the summary scores to inform interventions. However, when an individual reaches the ~2.5 level for closed spaces or the ~3 level for open spaces, clinicians may considering initiating early interventions as a proactive measure to prevent the onset of fear of falling and the development of avoidance behaviors.

When considering the magnitude of the factor loadings across the items, the transitional movements (i.e., chair to bed, sit to stand, vice-vera) had the greatest factor loading into the closed environment factor, indicating a strong relationship between these activities and the observed factor. Transitional movements require coordination between one’s muscles, balance, and sensory input. Muscle weakness, balance impairments, and sensory problems are often experienced by persons with MS (Cattaneo and Jonsdottir, 2009; Citaker et al., 2013), rendering these movements challenging. Though postural transitions are understudied in MS (Spain et al., 2012), research in older adults proposed that these movements offer a more reliable metric for assessing physical activity and are less influenced by environmental conditions compared to step count and walking duration (De Bruin et al., 2007), and research in stroke patients found that many falls occur when these individuals change position (Nyberg and Gustafson, 1995). Consequently, these movements may become potential triggers of FOF. Importantly, these transitional movements often must be performed multiple times during any given day (Parvaneh et al., 2017). As a result, an individual may either experience constant FOF during these common motor tasks or may restrict engaging in physical activity to minimize changing positions and performing these transitional movements. Addressing FOF in the context of transitional movements is crucial not only for fall prevention, but also for promoting functional independence in the MS community.

Interestingly, when examining the results of the EFA, the stairs item did not load well into either factor and was therefore removed from further analysis. The item read “walking up and down stairs” without any clarification regarding whether the stairs were inside the home or outdoors. We believe this lack of clarification resulted in this item not fitting well into the open or closed environment factor. Stairs within the home may be associated with a sense of familiarity, potentially making the act of walking up or down them less intimidating compared to outdoor stairs. The home environment provides a controlled and predictable setting in which the lighting and spatial layout align with the individual’s daily routine. This familiarity provides a level of comfort and confidence which may allow for the individual to focus more on the act of ascending/descending the stairs without being preoccupied with hazards that may be present on outdoor stairs (uneven surfaces, availability of handrails, varying degrees of elevation, ice, rocks, etc.). Therefore, the perceived risk of falling associated with stairs in the home may be considerably lower, and thus individuals may experience lower FOF when performing this activity compared to stairs outside of the home. Importantly, we acknowledge that walking up/down the stairs is a challenging activity that often results in falls and subsequent injuries or death (Hamel et al., 2005; Talbot et al., 2005; Lee and Chou, 2007; Verghese et al., 2008). Stairs are a common architectural feature used to transition between open and closed environments. The factor analysis cross-loading onto both factors supports this. Future studies interested in stairs should be more specific on the context (e.g., indoor vs. outdoor), or create multiple items similar to stairs (e.g., ramps, raised thresholds).

Summary scores from the open and closed environment factors were used to predict whether an individual only reported CAF compared to reporting both CAF and FOF, controlling for age and sex. Our findings revealed that age was not significantly associated with FOF, in line with previous work studying factors associated with FOF in patients with MS (Khalil et al., 2017). Females were nearly 2.5 times more likely to be classified as concerned + fearful compared to males. This finding is consistent with prior studies in healthy older adults, which have found that being female is a main risk factor for developing FOF (Scheffer et al., 2008; Lavedán et al., 2018), as well as research in MS, demonstrating that women are significantly more likely to report FOF compared to men (Peterson et al., 2007). Interestingly, another study evaluating differences between physiological and perceived fall risk found that individuals with low physiological fall risk who rated their fall risk excessively high were more likely to be female (Delbaere et al., 2010). Therefore, females may be more at risk for developing FOF due to psychological factors rather than motor (balance and walking) impairments, as females with MS report more anxiety than males (Jones et al., 2012), a mental health problem that is highly correlated with FOF (Brown et al., 2006; Tuerk et al., 2015).

Prior research has highlighted the lack of a consistent definition for FOF (Legters, 2002; Jung, 2008; Ellmers et al., 2023), often using the term interchangeably with related, but distinct concepts such as CAF, fall-related efficacy, balance confidence. In the absence of clear definitions, understanding how CAF and FOF may impact fall risk, behavior, and thought processes becomes challenging, emphasizing the need to clarify the impacts of the two constructs. We speculate that CAF involves a rational appraisal of potential risks and appropriate behavioral modifications to prevent falls; CAF is based on an accurate sense of awareness of one’s environment and physical limitations. In contrast, we believe that FOF involves an intense emotional response accompanied by heightened anxiety and stress, leading to avoidance behaviors (Zijlstra et al., 2007; Landers et al., 2011; Landers and Nilsson, 2023) and reduced activity participation (Peterson et al., 2007; Kalron et al., 2018). CAF may be a protective mechanism that prompts individuals to take precautionary measures to avoid falling, whereas FOF may significantly reduce one’s quality of life by restricting mobility and fostering a cycle of physical inactivity and functional decline (Figure 1). Elucidating the difference between these two psychological constructs is crucial for clinicians to tailor appropriate interventions, as an individual who experiences CAF may respond better to balance training, while an individual with FOF may need balance interventions and psychological treatment such as cognitive-behavioral therapy to address the heightened fear response (Peeters et al., 2020; Landers and Nilsson, 2023). In our sample, 60% of participants indicated that CAF is a separate experience than FOF, and previous work using focus groups revealed that individuals with MS consider concern and fear to be constructs that lie on a continuum (Matsuda and Hoffman, 2022). Future studies should seek to define these terms and the shift of CAF into FOF from the MS communities’ perspective.

### 4.1 Limitations

We acknowledge that our sample consisted of primarily of White female participants (Table 2); however, MS affects women disproportionally at a 3:1 female-to-male ratio (McGinley et al., 2021), and White individuals comprise the largest racial group impacted by MS, followed by Black individuals and Hispanic individuals, respectively (Hittle et al., 2023). Therefore, our sample is relatively representative of this clinical population. Additionally, the sample reported relatively low disability, with an average PDDS score of 3.01, limiting the ability to generalize our findings to individuals with MS with greater walking impairments. Further, our study solely relied on self-report measures. Though self-report studies offer many advantages, such as the ability to survey a large sample size, they also are associated with inherent limitations that we must consider when interpreting our results. For example, individuals may develop a response pattern in which they consistently select the same response option through many different questions (e.g., selecting the midpoint of the scale for each item), which would threaten the validity of the data. Additionally, the accuracy of self-report measures relies on the participant’s ability to recall past events and feelings accurately, which may be impaired in persons with MS as many individuals experience memory and cognitive dysfunctions (D’Orio et al., 2012; Rahn et al., 2012). Particularly important for pwMS who experience cognitive impairments, the scale in the CAFFE and the independent questions inquiring about CAF and FOF may not have been clearly understood, as these are complex constructs we did not provide definitions for. This potential lack of comprehension may be reflected by the ~17% of participants who responded “maybe” and “I do not know” to these questions. Additionally, participants’ mood, emotional state, or current environment may influence their responses. Importantly, there were no checks to ensure the responses came directly from the individual with MS and not a relative or caregiver. Finally, all data were collected cross-sectionally, making it challenging to determine changes over time or establish causal relationships. Future studies should consider combining self-report measures of CAF and FOF with physiological measures of fear to help mitigate some of these limitations and provide a more comprehensive understanding of these phenomenon.

### 4.2 Conclusion

This study aimed to develop a novel scale with sound psychometric properties to capture the shift from CAF to FOF. The findings here demonstrated that the CAFFE is an assessment tool with defined tipping points to evaluate CAF and FOF with sufficient internal consistency and structural validity in the MS community. Further, our results suggest that the majority of persons with MS consider CAF and FOF to be separate constructs that lie on a continuum, with CAF being more prevalent than FOF. Given these findings, the CAFFE offers clinicians an efficient way to assess CAF and FOF. Further research is needed to identify individuals with CAF who are at risk of tipping (or developing) into having FOF to drive targeted rehabilitation efforts and break the vicious FOF cycle. Future work will focus on developing short-form versions of the novel questionnaire to reduce repetitiveness across the items and reduce the assessment time, and will collect longitudinal data to evaluate predictive validity of the CAFFE.

## Data availability statement

The raw data supporting the conclusions of this article will be made available by the authors, without undue reservation.

## Ethics statement

The studies involving humans were approved by Wayne State University Institutional Review Board and University of Washington Institutional Review Board. The studies were conducted in accordance with the local legislation and institutional requirements. The participants provided their written informed consent to participate in this study.

## Author contributions

TT: Writing – original draft, Writing – review & editing PM: Conceptualization, Writing – review & editing. TH: Writing – review & editing. AD: Methodology, Supervision, Writing – review & editing. NF: Conceptualization, Supervision, Writing – review & editing.
